# Traditional Atlantic Diet and Its Effect on Health and the Environment: A Secondary Analysis of the GALIAT Cluster Randomized Clinical Trial

**DOI:** 10.1001/jamanetworkopen.2023.54473

**Published:** 2024-02-07

**Authors:** Cristina Cambeses-Franco, Francisco Gude, Alfonso J. Benítez-Estévez, Sara González-García, Rosaura Leis, Juan Sánchez-Castro, María Teresa Moreira, Gumersindo Feijoo, Mar Calvo-Malvar

**Affiliations:** 1 CRETUS Centre, Department of Chemical Engineering, School of Engineering, University of Santiago de Compostela, Spain; 2 Concepción Arenal Primary Care Center, Department of Family and Community Medicine, University of Santiago de Compostela, Health Research Institute of Santiago de Compostela, Spain; 3 Department of Laboratory Medicine, University Clinical Hospital of Santiago de Compostela, Spain; 4 Unit of Pediatric Gastroenterology, Hepatology and Nutrition, Pediatric Service, University Clinical Hospital of Santiago de Compostela, Spain; 5 A Estrada Primary Care Center, A Estrada, Pontevedra, Spain; 6 Health Research Institute of Santiago de Compostela, Spain

## Abstract

**Importance:**

The universal call to action for healthier and more sustainable dietary choices is the framework of the United Nations’s Sustainable Development Goals. The Atlantic diet, originating from the northwest of the Iberian Peninsula, represents an example of a traditional diet that aligns with these principles.

**Objective:**

To explore a 6-month intervention based on the Atlantic diet’s effects on metabolic and environmental health, assessing metabolic syndrome (MetS) incidence and the carbon footprint.

**Design, Setting, and Participants:**

The Galician Atlantic Diet study was a 6-month randomized clinical trial designed to assess the effects of this regional traditional diet on families’ eating habits. The study was conducted from March 3, 2014, to May 29, 2015, at a local primary health care center in the rural town of A Estrada in northwestern Spain and involved a multisectoral collaboration. Families were randomly selected from National Health System records and randomized 1:1 to an intervention or control group. This secondary analysis of the trial findings was performed between March 24, 2021, and November 7, 2023.

**Interventions:**

Over 6 months, families in the intervention group received educational sessions, cooking classes, written supporting material, and foods characteristic of the Atlantic diet, whereas those randomized to the control group continued with their habitual lifestyle.

**Main Outcomes and Measures:**

The main outcomes were MetS incidence, defined per National Cholesterol Education Program Adult Treatment Panel III guidelines, and carbon footprint emissions as an environmental metric using life cycle assessment with daily dietary intake as the functional unit.

**Results:**

Initially, 250 families were randomized (574 participants; mean [SD] age, 46.8 [15.7] years; 231 males [40.2%] and 343 females [59.8%]). The intervention group included 126 families (287 participants) and the control group, 124 families (287 participants). Ultimately, 231 families completed the trial. The intervention significantly reduced the risk of incident cases of MetS (rate ratio, 0.32; 95% CI, 0.13-0.79) and had fewer MetS components (proportional odds ratio, 0.58; 95% CI, 0.42-0.82) compared with the control condition. The intervention group did not have a significantly reduced environmental impact in terms of carbon footprint emissions compared with the control group (−0.17 [95% CI, −0.46 to 0.12] kg CO_2_ equivalents/person/d).

**Conclusions and Relevance:**

These findings provide important evidence that a family-focused dietary intervention based on a traditional diet can reduce the risk of incident MetS. Further research is needed to understand the underlying mechanisms and determine the generalizability to other populations, taking into account regional cultural and dietary variations.

**Trial Registration:**

ClinicalTrials.gov Identifier: NCT02391701

## Key Points

**Question:**

Could traditional dietary patterns effectively address the complex relationship between environmental sustainability and the incidence of diet-related diseases like metabolic syndrome?

**Findings:**

In this secondary analysis of a cluster randomized clinical trial including 574 participants, a traditional Atlantic dietary intervention significantly reduced the incidence of metabolic syndrome. There was no statistically significant difference in the reduction in dietary carbon footprint emissions in the intervention group compared with the control group.

**Meaning:**

These findings suggest that traditional diets could serve as valuable tools to promote the convergence of human and planetary health, making them noteworthy models of sustainable and healthy dietary patterns.

## Teaser

This secondary analysis of a cluster randomized clinical trial assesses the effects of the Atlantic diet on metabolic syndrome incidence and the environmental carbon footprint.

## Introduction

Diets worldwide are changing, often with a negative impact on human and planetary health. To achieve the United Nations’s Sustainable Development Goals (SDGs) on noncommunicable disease reduction (SDG 3) and climate change mitigation (SDG 13), a shift to sustainable, healthy dietary patterns is necessary.^1^ These diets should support health and be environmentally friendly, accessible, affordable, safe, equitable, and culturally acceptable.^1^ Some traditional regional diets, such as the Atlantic diet, align with this concept. The Atlantic diet shares similarities with the Mediterranean diet and has been linked to lower metabolic risk factors^2,3,4,5,6^ and environmental benefits.^7^

We hypothesize that a shift to a traditional Atlantic diet would not only improve population health but also contribute to the sustainability of the environment, while taking into account the identity and diversity of the food system and culture of northwest Spain and northern Portugal. To date, no clinical trial has been conducted that assesses the effects of a traditional diet (ie, the Atlantic diet) from a dual-sustainability perspective on human and environmental health, thus addressing 2 of the United Nations’s SDGs. Therefore, in this report, we address 2 questions: (1) whether an intervention based on a traditional Atlantic diet can reduce the incidence of new cases of metabolic syndrome (MetS) or contribute to a higher resolution of existing cases compared with a general population and (2) whether this intervention would result in a lower environmental impact.

## Methods

### Study Design

This study is a post hoc secondary analysis of the Galicia Atlantic Diet (GALIAT) study, a 6-month, community-focused randomized clinical trial designed to assess the effects of the traditional Atlantic diet on metabolic health and dietary habits in families. The GALIAT trial was performed from March 3, 2014, to May 29, 2015, at the local primary health care center in the rural town of A Estrada in northwestern Spain. This trial was grounded in the traditional diet of the study area and entailed a collaborative, multisectoral approach involving citizens, local businesses, researchers, and public institutions.^8^ The trial was deliberately shaped to be pragmatic, prioritizing effectiveness and practicality in primary care settings.^9^

Study procedures are detailed in previous publications,^6,8^ and the study protocol is provided in Supplement 1. The Galacian Autonomic Committee for Research Ethics approved the trial (code 2013/531) and the current study. During the preassessment session, participants were briefed on the project’s objectives, informed about subject selection methods, given detailed information on participation requirements, and acquainted with their rights as participants. Additionally, comprehensive project documentation, along with informed consent forms for all family members (including adults, children, and guardians) were provided. Participants who chose to participate further were then scheduled for an initial evaluation at the health center where, accompanied by their families, they submitted signed informed consent forms and underwent evaluations to confirm compliance with the inclusion/exclusion criteria. The trial was performed in accordance with the Declaration of Helsinki and the principles of Good Clinical Practice. The study was retrospectively registered with ClinicalTrials.gov (NCT02391701) and followed the Consolidated Standards of Reporting Trials (CONSORT) 2010 guidelines^10^ and the Template for Intervention Description and Replication (TIDieR) checklist.^11^ The current data analysis was performed between March 24, 2021, and November 7, 2023.

### Participants

Recruitment within the community was performed through a random representative sample of 3500 individuals, aged 18 to 85 years and stratified by decades of age, drawn from the Spanish National Health System Register of a single rural population of approximately 20 000 inhabitants. These index individuals and their household family members were invited to participate if they lived in a family unit of at least 2 members aged 3 to 85 years; were not currently taking lipid-lowering medication; were not pregnant; and did not have alcoholism,^12^ major cardiovascular disease, dementia, or a life expectancy of less than 1 year, which specifically refers to individuals with a terminal disease (Supplement 1). The study comprised participants of Spanish ethnicity and Caucasian descent. Fieldwork personnel gathered data on the specific birthplaces and family histories of the participants.

### Randomization and Procedures

Families were randomly assigned in a 1:1 ratio to the intervention or control group using a computer-generated random number table. The dietary intervention was based on the Atlantic diet, the traditional diet in northwestern Spain and Portugal, which is composed of local, fresh, and minimally processed seasonal foods like vegetables, fruits, whole grains, beans, and olive oil. The Atlantic diet also features high fish and seafood consumption, along with starch-based products, dried fruits (particularly chestnuts), milk, cheese, and moderate meat and wine intake.^8,13^

The dietary counseling aimed to modify food habits in accordance with the characteristics of the Atlantic diet but not necessarily to restrict energy intake. Dietary recommendations were adapted to conform to the preferences and nutritional needs of each participant. In the intervention group, families attended 3 nutrition education sessions at the health care center and received additional support, including a cooking class, written materials, and regular food baskets with traditional Atlantic diet items (eFigure 1 in Supplement 2). Control group participants were advised to maintain their usual lifestyle. Previous articles assessed dietary patterns, with baseline values showing similarity (eMethods in Supplement 2).^9^

At the baseline and after 6 months, information was collected on dietary intake, physical activity,^14^ medication use, and other variables. Masking procedures were implemented at the randomization and data entry stages to minimize potential biases. However, due to the nature and design of our study, masking at the intervention level was not feasible.

### Outcomes

#### Definition of MetS

Metabolic syndrome was defined according to criteria from the National Cholesterol Education Program Adult Treatment Panel III (ATP III) guidelines^15^ and based on meeting at least 3 of the following 5 criteria: (1) waist circumference greater than 102 cm for men or greater than 88 cm for women, (2) triglyceride levels of 150 mg/dL or higher (≥1.7 mmol/L), (3) high-density lipoprotein cholesterol less than 40 mg/dL (<1.03 mmol/L) for men or less than 50 mg/dL (<1.3 mmol/L) for women, (4) blood pressure of 130/85 mm Hg or higher, and (5) fasting glucose level of 110 mg/dL or higher (≥6.2 mmol/L). Participants who were being treated with antidiabetic, antihypertensive, or triglyceride-lowering medications were classified as positive for the respective criterion. We investigated (1) whether the intervention can reduce the incidence of new cases (among those participants without MetS at baseline) and (2) whether there were differences in the prevalence of MetS and its components (among all-comers) at the end of follow-up between the intervention and control groups.

#### Metabolic Variables

Metabolic variables were measured at the primary health care center. All anthropometric measurements were made in triplicate (eMethods in Supplement 2).

#### Dietary Intake

A 3-day food diary was used to collect dietary intake data (eMethods in Supplement 2). Nutritionists verified all records completed by participants in their presence. Dietary intake was analyzed using DIAL, version 3.3.5.0 professional nutrition analysis software.^16^

### Carbon Footprint

#### Life Cycle Assessment

Life cycle assessment (LCA) is a method of quantifying the environmental impacts of the life cycle of products, processes, or services.^17^ The most common environmental impact category assessed in LCA is the carbon footprint, the best-known indicator of climate change.

#### Functional Unit

The LCA approach was used to calculate each participant’s dietary carbon footprint, relative to a functional unit, as a baseline for all calculations. The functional unit chosen was individual daily dietary intake based on the results of the dietary questionnaires.

#### Methodology

The carbon footprint framework assessed CO_2_ emissions associated with each participant’s diet. This well-established indicator was used while considering variations in LCA study boundaries. Our approach focused on the life cycle from food production to household consumption, with adjustments for supply chain food losses and waste (eMethods and eFigure 2 in Supplement 2).^18,19,20,21,22,23^

### Statistical Analysis

Sample size was determined for the primary outcome (Supplement 1). Analyses were conducted for the intention-to-treat population, with missing data imputed using the multivariable imputation by chained equations method.^24^ The main analysis used multiple imputation using Markov chain Monte Carlo method with 30 imputations for each missing measurement. A per-protocol sensitivity analysis was also performed. Baseline comparability was assessed using χ^2^ tests (categorical variables) and Student *t* tests (continuous variables). Modified Poisson regressions with robust error variance were used to estimate rate ratios (RRs) and their 95% CIs for new MetS cases (in participants without MetS) in the intervention group compared with the control group^25^ after adjusting for age and sex. For exploratory purposes, point prevalences (with 95% CIs) for MetS and its components at baseline and after 6 months were estimated.

Cumulative logit regression models for ordered categories were constructed to examine the effect of the intervention on the number of MetS risk factors as an ordinal variable ranked from 0 to 5 compared with control condition. Mixed-effects linear models were used to evaluate changes in carbon footprint emissions from baseline to the 6-month follow-up in all randomized participants, as well as in those who completed the study. These models were adjusted for age, sex, and baseline values, with intervention as a fixed effect and family as the random effect. The intraclass correlation coefficient (ICC) was calculated to examine how much variance in the carbon footprint outcome was explained by the family cluster effect. All analyses were conducted using Stata, version 16.0 statistical software (StataCorp LLC). The χ^2^ test was used for differences between categorical variables, and the Student *t* test for continuous variables. All statistical tests were 2-sided, and *P* < .05 was deemed statistically significant.

## Results

### Baseline Characteristics

**Figure 1.**
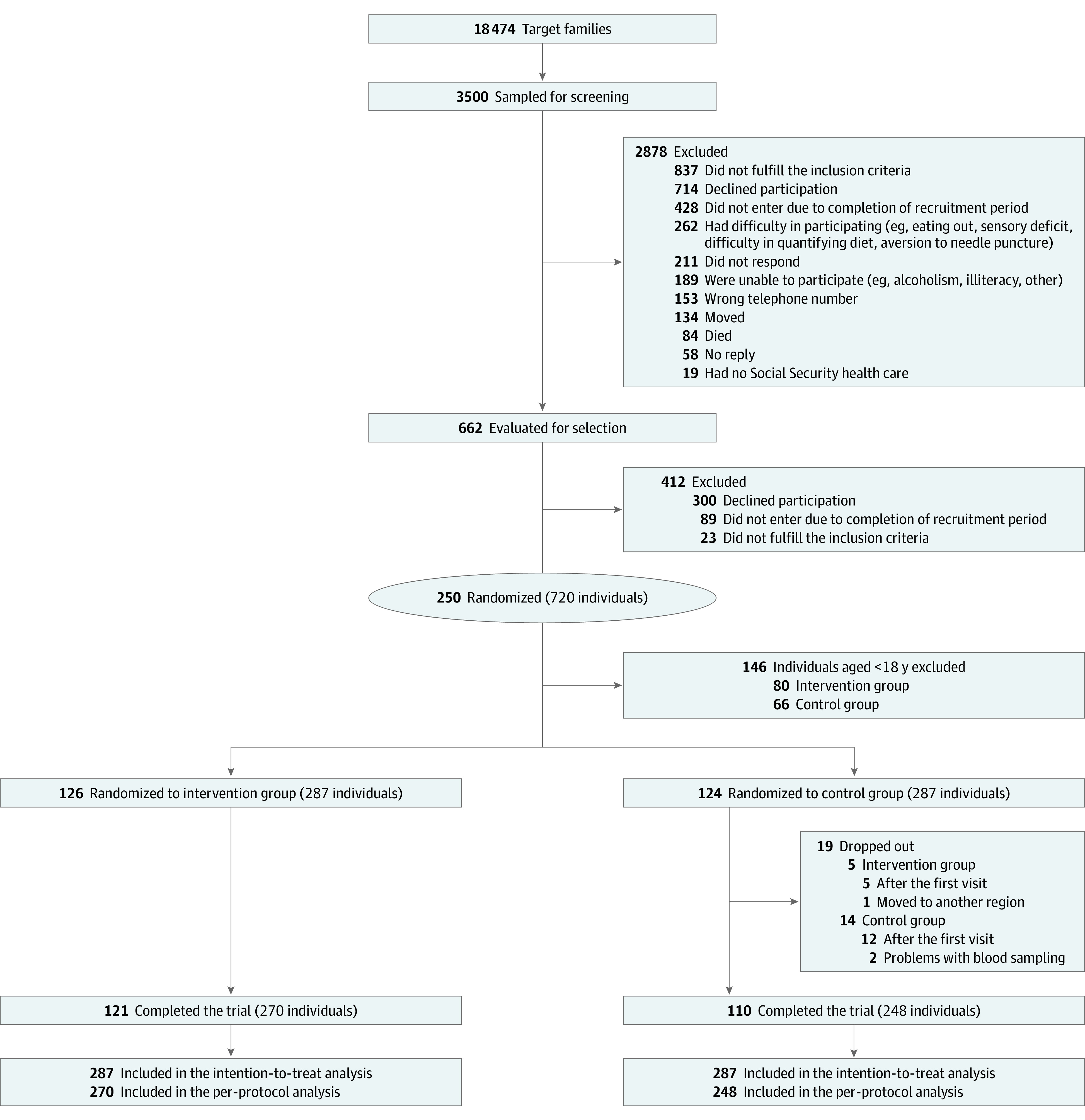
Overview of the Study Population

Of 250 families (720 participants) who were recruited and randomized, a total of 121 families (270 adults [aged ≥18 years]) in the intervention group and 110 (248 adults) in the control group completed the trial (Figure 1). Briefly, all participants were White, the mean (SD) number of persons per family unit was 2.3 (0.8), the mean (SD) participant age was 46.8 (15.7) years, and 231 participants (40.2%) were male and 343 (59.8%) female. As previously reported,^6^ the randomization produced 2 groups with similar characteristics of baseline sociodemographic and potential confounding factors, including education, smoking, alcohol intake, and physical activity, with the exception of age, which was significantly higher in the intervention group (mean [SD], 48.2 [15.8] vs 45.3 [15.4] years for the intervention and control groups, respectively) (Table 1). All individuals in the intervention group participated in the nutrition education program. A total of 241 of 287 (84.0%) participants in the intervention arm and 238 of 287 (82.9%) in the control group filled out the 3-day food record completely at baseline and 6 months.

**Table 1.** Baseline Characteristics of the Participants in the Study

Characteristic	No. (%)^a^	
	Control group	Intervention group
No. of participants		
Families	124	126
Individuals	287	287
Participants per family, mean (SD)	2.3 (0.8)	2.3 (0.7)
Sex		
Male	114 (39.7)	117 (40.8)
Female	173 (60.3)	170 (59.2)
Age, mean (SD), y	45.3 (15.4)	48.2 (15.8)
Marital status		
Married or with partner	195 (67.8)	211 (73.4)
Divorced, separated, or widowed	33 (11.5)	28 (9.8)
Single	59 (20.6)	48 (16.8)
Educational level		
None	29 (10.1)	30 (10.5)
Elementary	120 (41.8)	103 (35.9)
Secondary	91 (31.7)	103 (35.9)
University or higher	47 (16.4)	51 (17.7)
Employment status		
Employed	148 (51.6)	139 (48.4)
Retired	40 (13.9)	56 (19.5)
Other^b^	99 (34.5)	92 (32.1)
Smoking status		
Never	128 (44.6)	120 (41.8)
Former	50 (17.4)	71 (24.7)
Current	109 (38.0)	96 (33.5)
Alcohol intake		
Abstinent	125 (43.5)	124 (43.2)
Light (1-140 g/wk)	134 (46.6)	130 (45.3)
Heavy (>140 g/wk)	28 (9.9)	33 (11.5)
Comorbidities		
Cardiovascular disease	42 (16.0)	49 (18.3)
Cerebrovascular accident	3 (1.1)	3 (1.1)
Diabetes	16 (5.9)	16 (5.9)
Current medications		
Cholesterol lowering	23 (8.7)	32 (12.5)
Antihypertensives	44 (18.1)	56 (24.2)
International Physical Activity Questionnaire		
Inactive	56 (19.8)	44 (15.3)
Minimally active	68 (24.6)	85 (29.6)
Active	163 (55.6)	158 (55.1)

^a^ No significant differences were found between the 2 groups, with the exception of age, which was significantly higher in the intervention group (χ^2^ test for differences between categorical variables and Student *t* test for continuous variables).

^b^ The other category accounts for diverse occupational situations not explicitly classified under employed or retired (eg, students, subsistence farmers, individuals with disabilities).

### Effect of the GALIAT Intervention on MetS and Its Components

Of the 457 participants without MetS at the beginning of the trial, 23 developed MetS during the 6-month follow-up (6 [2.7%] in the intervention group; 17 [7.3%] in the control group). There was a significant reduction in incident MetS cases for the intervention group (RR, 0.32; 95% CI, 0.13-0.79) compared with the control group (Table 2).

**Table 2.** Incidence Rate, Prevalence Rate, and Rate Ratios (RRs) of Metabolic Syndrome (MetS) and Its Components by Treatment Group^a^

Criterion^b^	% (95% CI)				RR (95% CI)^c^	*P* value
	Control group (n = 287)		Intervention group (n = 287)			
	Baseline	6 mo	Baseline	6 mo		
Abdominal obesity	40.1 (34.5-45.8)	41.8 (36.1-47.5)	51.9 (46.1-57.7)	47.0 (41.3-52.8)	0.90 (0.82-1.00)	.04
Hypertriglyceridemia	18.5 (14.0-23.0)	17.4 (13.0-21.8)	13.9 (9.9-18.0)	13.9 (9.9-18.0)	0.95 (0.69-1.30)	.74
Low HDL cholesterol	18.8 (14.3-23.3)	27.2 (22.0-32.3)	19.2 (14.6-23.3)	21.6 (16.8-23.7)	0.79 (0.64-0.97)	.03
High blood pressure	40.8 (35.1-46.5)	42.9 (37.1-48.6)	49.8 (44.0-55.6)	44.3 (38.5-50.0)	0.87 (0.76-1.00)	.05
Hyperglycemia	17.4 (13.0-21.8)	15.0 (10.8-19.1)	17.4 (13.0-21.8)	13.9 (9.9-18.0)	0.92 (0.69-1.23)	.56
MetS prevalence rate	18.8 (14.2-23.3)	19.2 (14.6-23.7)	22.0 (17.2-26.8)	17.8 (13.3-22.2)	0.82 (0.64-1.06)	.13
MetS incidence rate	NA	7.3 (3.9-10.6)	NA	2.7 (0.6-4.8)	0.32 (0.13-0.79)	.01

Abbreviations: HDL, high-density lipoprotein; NA, not applicable.

^a^ Values used from the intention-to-treat data set.

^b^ Criteria from the National Cholesterol Education Program Adult Treatment Panel III for the definition of MetS are as follows: abdominal obesity (waist circumference >102 cm in males or >88 cm in females), hypertriglyceridemia (fasting serum triglycerides ≥150 mg/dL), low HDL cholesterol levels (fasting HDL cholesterol <40 mg/dL in males or <50 mg/dL in females), high blood pressure (≥130/85 mm Hg or current antihypertensive medication use), and hyperglycemia (fasting blood glucose ≥110 mg/dL or current antidiabetic therapy). Individuals meeting at least 3 of these criteria were considered to have MetS.

^c^ Adjusted for sex and age and estimated using Poisson regression models for incident cases of MetS per 6 mo.

**Figure 2.**
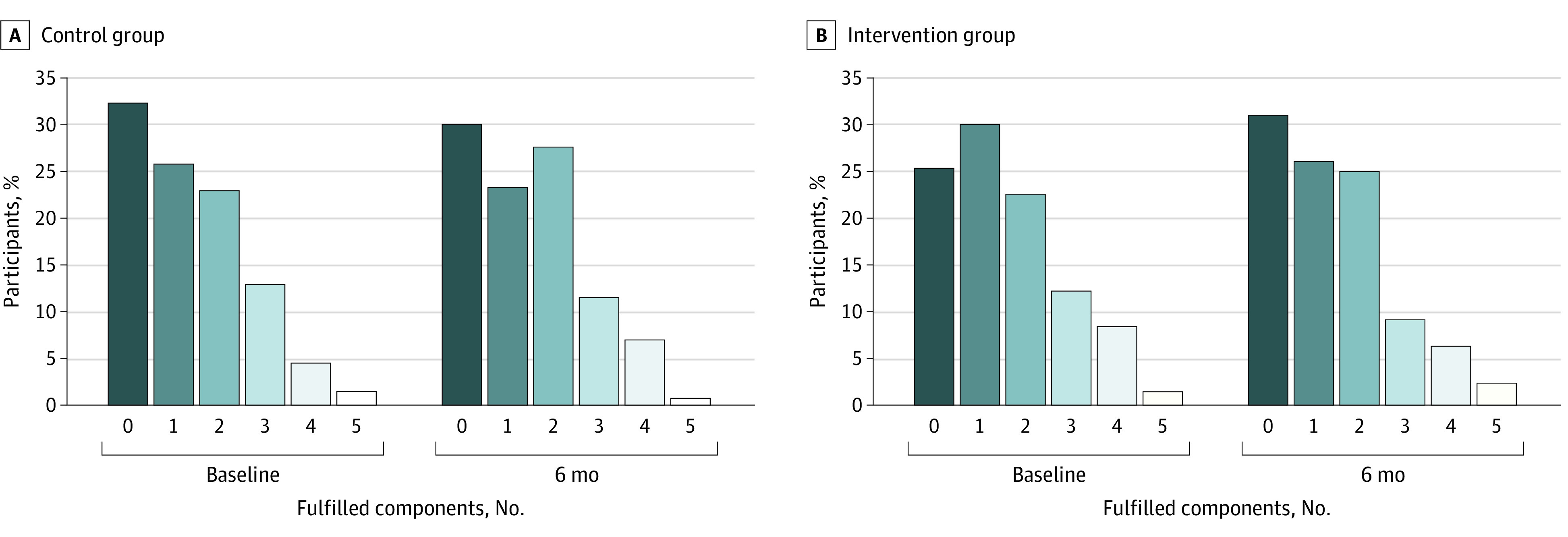
Distribution of Metabolic Syndrome Component Score by Treatment Group

Initially, 117 participants (20.4%) met the ATP III criteria for MetS, with 63 (22.0%) in the intervention group and 54 (18.8%) in the control group (Figure 2). After the 6-month follow-up, 18 participants in the intervention group (28.6%) and 16 (29.6%) in the control group no longer met these criteria. Therefore, in the whole sample (eg, participants with and without MetS), the intervention was not significantly associated with a reduced risk in overall MetS prevalence (RR, 0.82; 95% CI, 0.64-1.06) (Table 2). The most common MetS features were abdominal obesity (46.0%; 95% CI, 41.9%-50.1%) and high blood pressure (45.3%; 41.2%-49.4%). The intervention group saw a significant decrease in waist circumference (mean [SD] change, −1.79 [0.40] cm; *P* < .001) but no significant decreases in blood pressure (systolic: mean [SD] change, −1.4 [1.0] mmHg [*P* = .17]; diastolic: mean [SD] change, −0.72 [0.64] mmHg [*P* = .27]).

For individual MetS components, the intervention reduced the risk of central obesity (RR, 0.90; 95% CI, 0.82-1.00) and low high-density lipoprotein cholesterol (RR, 0.79; 95% CI, 0.64-0.97) compared with the control condition (Table 2). The intervention had no significant effect on high blood pressure, high triglyceride levels, or high fasting serum glucose levels compared with the control condition. Additional modeling details are provided in eTable 5 in Supplement 3. Per-protocol analysis showed similar results, with the exception of a significant reduction in hypertension risk associated with the intervention (eTable 1 in Supplement 2).

Multivariable ordered logistic regression analysis revealed that participants in the intervention group had a proportional odds ratio of 0.58 (95% CI, 0.42-0.82) for having more MetS components compared with those in the control group (Table 3), which means that those in the intervention group were approximately 42% less likely to exhibit an additional MetS component compared with the control group. Sensitivity analysis using the per-protocol population showed similar results in magnitude and direction (eTable 2 in Supplement 2).

**Table 3.** Distribution of Metabolic Syndrome (MetS) Component Score by Treatment Group Among 574 Participants

No. of fulfilled components of MetS	Control group (n = 287)		Intervention group (n = 287)		POR (95% CI)^a^	*P* value
	Baseline	6 mo	Baseline	6 mo		
0	0.32 (0.27-0.38)	0.30 (0.25-0.35)	0.25 (0.20-0.31)	0.31 (0.26-0.36)	0.58 (0.42-0.82)	.002
1	0.26 (0.21-0.31)	0.23 (0.18-0.28)	0.30 (0.25-0.35)	0.26 (0.21-0.31)		
2	0.23 (0.18-0.28)	0.28 (0.22-0.33)	0.23 (0.18-0.28)	0.25 (0.20-0.30)		
3	0.13 (0.09-0.17)	0.12 (0.08-0.15)	0.12 (0.08-0.16)	0.09 (0.06-0.12)		
4	0.05 (0.02-0.07)	0.07 (0.04-0.10)	0.08 (0.05-0.12)	0.06 (0.04-0.09)		
5	0.01 (0.00-0.03)	0.01 (0.00-0.02)	0.01 (0.00-0.03)	0.02 (0.01-0.04)		

Abbreviation: POR, proportional odds ratio.

^a^ Ordered logistic regression modeling was performed to estimate the POR and 95% CI, adjusted for age and sex.

### Effect of the GALIAT Intervention on Carbon Footprint Emissions

After 6 months, the carbon footprint score was reduced in the control group (baseline: mean [SD], 3.71 [1.55] kg CO_2_ equivalents [kgCO_2_eq]/person/d; after 6 months: mean [SD], 3.56 [1.50] kgCO_2_eq/person/d) and the intervention group (baseline: mean [SD], 3.60 [1.44] kgCO_2_eq/person/d; after 6 months: mean [SD], 3.38 [1.39] kgCO_2_eq/ person/d) (eTable 3 and eFigure 3 in Supplement 2). Linear mixed regression analysis did not show a significant between-group difference (−0.17 kgCO_2_eq/person/d; 95% CI, −0.46 to 0.12), after adjusting for baseline values, age, and sex and with family as the random effect. The sensitivity analysis for the carbon footprint using the per-protocol population showed similar results (eTable 4 in Supplement 2). The ICC revealed that approximately 45% of the variability in the carbon footprint score was due to family membership, revealing the importance of the family belongingness in the emission of greenhouse gases associated with food.

## Discussion

In a community-based sample from the GALIAT randomized clinical trial, we jointly examined the effects of a nutritional intervention based on a traditional Atlantic diet on MetS and associated changes in its components and the environmental outcomes associated with the Atlantic diet in terms of carbon footprint. Our main finding was that the 6-month nutritional intervention showed a lower risk of developing MetS among participants in the intervention group compared with the control group. Furthermore, individuals in the intervention group were approximately 42% less likely to exhibit an additional MetS component than those in the control group, although there was no evidence of a reduction in the RRs of high blood pressure, hypertriglyceridemia, or hyperglycemia, separately. Finally, the comparison between food consumption in the intervention and control groups did not achieve a statistically significant reduction in environmental impact.

Our study provides important contributions to the field. First, the positive outcomes for MetS and carbon footprint emissions were observed in a community-based sample following a diet that aligns with their cultural heritage. Second, the evidence provided for a randomized clinical trial that includes environmental parameters offers unique and valuable insight into how traditional diets can promote both health outcomes and environmental sustainability in line with the United Nations’s 2030 Agenda.^26^ Finally, the ICCs indicate that the cluster (family) factor had an important influence on the mean change of greenhouse gas emissions, supporting the effectiveness of family-based approaches for mitigating the impact of food-related greenhouse gas emissions.

It is well established that weight loss has a great benefit for the treatment of all the components of the MetS, which are excessive adiposity, dyslipidemia, hypertension, insulin resistance, and hyperglycemia.^27,28,29,30^ Approximately 20% of the study participants had MetS at baseline, consistent with the prevalence observed in a large study of 24 670 individuals from the general Spanish population, aged 35 to 74 years, without diabetes or cardiovascular disease.^31^ Similarly, our study’s findings on abdominal obesity closely match the 51% prevalence reported in a sample of 17 980 individuals aged 18 to 80 years in Spain according to ATP III criteria.^32^

On the other hand, our study is also critical to understanding the implications of dietary choices on environment-related SDGs, especially SDG 13 (health and climate action). This relationship was shown through the randomized controlled nutritional intervention, where we observed a reduction of 0.17 kgCO_2_eq/person/d in the intervention group compared with the control group. We believe that the lack of statistical significance can be attributed to the study’s limited statistical power, since the sample size was primarily determined to detect changes in cholesterol levels (Supplement 1). We have estimated that a study with approximately 2000 participants would be necessary for this reduction to reach statistical significance with a *P* < .05. Therefore, the observed outcome supports our expectation that adherence to the Atlantic diet in the broader target population could significantly enhance the carbon footprint metric, contributing to efforts to achieve net-zero CO_2_ emissions by 2050 in line with SDG 13.^33^

These findings are broadly consistent with research showing that the traditional Atlantic diet is a climate-friendly diet, well ranked under environmental criteria.^7^ Moreover, results from previous work that mainly focused on carbon footprint emissions of the Atlantic diet based on bibliographic recommendations^7,18^ fall within the range of our average carbon footprint calculated for the intervention group after 6 months, taking into consideration associated deviations. For example, González-García et al^7^ designed a dietary scenario based on serving sizes as defined by the Spanish Society of Community Nutrition and data on current food consumption in Galician households and obtained a carbon footprint for the Atlantic diet of 3.62 kgCO2eq/person/d.

### Strengths and Limitations

This study has several strengths, including a randomized design, high retention rates, objective clinical and environmental measures, and a representative random sample from the general population. Our data offer valuable clinical insight into MetS and its prevention in a population-based context. We intentionally chose a community with moderate socioeconomic and educational levels to enhance generalizability, aligning with Organisation for Economic Co-operation and Development indicators at the time of study design.^34^ Baseline data suggest minimal bias, as randomization resulted in only slight group differences.

The study also has several limitations. Various unknown factors aside from the dietary intervention could have influenced clinical outcomes. In addition, the study intervention was complex, and it is not possible to determine which actions of the intervention may have contributed to the results. Thus, unmeasured aspects may exist. Contamination bias might be present, as the study received media attention, potentially influencing individuals’ lifestyles. The strategy of providing food baskets served as an incentive for session attendance and adherence but may limit generalizability to populations with food access challenges. The broad variety of carbon footprint emissions usually reported in published LCAs of food products, together with the diversity of food items in participant data, could have contributed to the environmental outcome. Moreover, 6 months may not have been long enough to properly assess metabolic changes. Follow-up of participants over a number of years could strengthen our results. Finally, our study’s sample size might lack the statistical power required to detect a significant reduction in CO_2_ emissions, indicating a potential need for larger-scale studies.

## Conclusions

As far as we are aware, the GALIAT study is the first community-based randomized clinical trial that, taking into account the identity and diversity of the region’s food system and culture, has shown the positive effect of a traditional diet intervention on the incidence of new cases of MetS, as well as on limiting greenhouse gas emissions. Our findings provide important evidence for the potential of traditional diets to accelerate progress toward achieving SDGs. Further research is needed to thoroughly understand the underlying mechanisms behind the observed outcomes and to determine the generalizability of these findings to other populations, taking into account the cultural and dietary variations of each region.

## Notes

Supplement 1.
Trial Protocol

Supplement 2.
eMethods.
**eFigure 1.** Intervention Components **eFigure 2.** System Boundaries Considered in the Carbon Footprint Assessment **eTable 1. **Incidence Rate, Prevalence Rate, and Rate Ratios of Metabolic Syndrome and Its Components by Treatment Group (Per-Protocol Data set) **eTable 2. **Distribution of MetS Component Score by Treatment Group (Per-Protocol Data set) **eFigure 3.** Frequency Distribution of Carbon Footprint at Baseline **eTable 3.** Differences in Dietary Carbon Footprint Between the Intervention and Control Groups (Intention-to-Treat Data set) **eTable 4. **Differences in Dietary Carbon Footprint Between the Intervention and Control Groups (Per-Protocol Data set)
eReferences

Supplement 3. **eTable 5.** Carbon Footprint (CF) per Foodstuff and Their Corresponding Source, Origin, and Percentages of Food Loss and Food Waste
eReferences

Supplement 4.
Data Sharing Statement
