# Supplementary material for: Traditional Atlantic Diet and Its Effect on Health and the Environment: A Secondary Analysis of the GALIAT Cluster Randomized Clinical Trial
Source: JAMA Netw Open. 2024 Feb 7;7(2):e2354473. doi: 10.1001/jamanetworkopen.2023.54473 (PMC10851095; doi:10.1001/jamanetworkopen.2023.54473)
Supplement: Supplement 1. — Trial Protocol [file jamanetwopen-e2354473-s001.pdf]

SP v 1.3 Approved: 20/12/2015

Study Protocol

1 TABLE OF CONTENTS

1 TABLE OF CONTENTS 1
2 ADMINISTRATIVE INFORMATION 3
2.1 Title and registration 3
2.2 Roles and responsibility 4
3 ABBREVIATIONS AND DEFINITIONS 5
4 INTRODUCTION 6
4.1 Background and rationale 6
4.2 Objectives 6
4.2.1 Study objectives 6
4.2.2 Research hypothesis 6
5 TRIAL METHODS 7
5.1 Trial design 7
5.2 Project coordination and location 8
5.3 Dietary intervention 9
5.2 Randomization 9
5.3 Sample size and power estimate 9
5.4 Framework 9
5.5 Statistical Interim analyses and trial halting 9
5.6 Timing of outcome assessments 10
6 STATISTICAL PRINCIPLES 13
6.1 Confidence intervals and *P* values 13
6.2 Adherence and protocol deviations 14
6.3 Analysis populations 14
7 TRIAL POPULATION 14
7.1 Screening data 14
7.2 Eligibility 14
7.3 Recruitment 15

7.4 Withdrawal/loss to follow-up 16
7.5 Baseline patient characteristics 16
8 ANALYSIS 17
8.1 Outcome definitions 17
8.1.1 Primary outcome 17
8.1.2 Secondary outcomes 17
8.1.3 Specific measurement and units 17
8.1.4 Calculations or transformations used to derive outcomes 17
8.2 Data validity and outliers 18
8.2.1 Normality 18
8.2.2 Identifying outliers 18
8.3 Missing data 18
8.4 Adverse effects 19
8.5 Statistical software 19
9 REFERENCES 19

2 ADMINISTRATIVE INFORMATION

2.1 TITLE, REGISTRATION, VERSIONS AND REVISIONS

**Official Title**	Study protocol for the GALIAT study: a population-based, cluster-randomized, controlled trial to assess the effects of a dietary intervention promoting the traditional Atlantic diet, conducted in the primary healthcare setting and with a community focus.
**Brief Title**	Study protocol for the GALIAT Study
**Acronym**	SP-GALIAT
**Local project number**	ITC-20133014
**Clinicaltrials.gov number**	NCT02391701
**Study protocol version**	This document was written based on the information contained in the documents listed below:<br>- Research Project submitted for approval to the European Regional Development Funds (ERDF-INNTERCONECTA for Galicia, Proposal 2013) 4 April 2013;<br>- Research Project submitted for approval to the European Regional Development Funds (ERDF-INNTERCONECTA for Galicia, Proposal 2015) 29 June 2015; and<br>- The research protocol submitted for approval to the Galician Autonomic Committee for Research Ethics, 5 November 2013.

2.2 ROLES AND RESPONSIBILITY

**Contributors and roles**	The team involved in the project includes Mar Calvo-Malvar [1], who has contributed to the design of the research protocol and written the original version of the study protocol, and will be responsible for coordinating the field work. Francisco Gude [2] has designed both the study and statistical protocols, and will be responsible for their correct application and validation of the results. Alfonso J. Benítez-Estévez [1] will oversee the databases and application of the statistical protocol, while Rosaura Leis [3] and Juan Sanchez-Castro [4] have contributed to the design of the research protocol and procedures, with Leis coordinating the nutritional protocol.
**Affiliations**	[1] Department of Laboratory Medicine, University Clinical Hospital of Santiago de Compostela, 15706, Santiago de Compostela, Spain<br><br>[2] Clinical Epidemiology and Biostatistics Unit, University Clinical Hospital of Santiago de Compostela, 15706, Santiago de Compostela, Spain<br><br>[3] Unit of Pediatric Gastroenterology, Hepatology and Nutrition, Pediatric Service, University Clinical Hospital of Santiago de Compostela, 15706, Santiago de Compost4ela, Spain.<br><br>[4] A Estrada Primary Care Center, A Estrada, 36680, Pontevedra, Spain
**Funding**	This project received funding from the ERDF-Innterconecta for Galicia Program - ITC-20133014 (2013 proposals) and ITC-20151009 (2015 proposals) - managed by the *Centro para el Desarrollo Tecnológico Industrial* (CDTI) (part of Spain's Ministry of Economy and Competitiveness.

3 ABBREVIATIONS AND DEFINITIONS

AUDIT-C	AUDIT alcohol consumption questions
BMI	Body Mass Index
CONSOT	Consolidated Standards of Reporting Trials
ERDF	European Regional Development Fund
FFQ	Food frequency questionnaire
HbA1c	Glycated hemoglobin
HDL cholesterol	High-density lipoprotein cholesterol
HOMA-IR	Homeostatic model assessment of insulin resistance
IPAQ-short	International Physical Activity Questionnaire (short format)
LDL cholesterol	Low-density lipoprotein cholesterol
SF-12v2	Spanish v.2.0 of the Short Form 12 Health Survey

4 INTRODUCTION

4.1 BACKGROUND AND RATIONALE

Diet is a major risk factor for a variety of chronic diseases, including cardiovascular disease, cancer, diabetes and other obesity-related conditions. For the prevention and control of lifestyle-related chronic diseases, the WHO recommends the strengthening of primary healthcare and the training of personnel working in this setting. However, improving the food habits of a community requires both a medical and social focus; population-based, multidisciplinary and culturally relevant interventions are needed. It was hypothesized that, from a public health point of view, an approach involving primary healthcare providers and the community itself would allow multilevel synergistic interventions aiding in the prevention and treatment of diet-related disease.

4.2 OBJECTIVES

4.2.1 Study objectives

The general objective of this trial is to examine the effectiveness of a community-focused dietary intervention, conducted in the primary healthcare setting and involving the traditional Atlantic diet, on the metabolic profile, anthropometric variables and nutritional habits of families.

The specific objectives would be to determine the effect of the intervention on (1) the lipid profile (2) anthropometric measurements and adiposity, (3) glucose metabolism and inflammation makers, and (4) dietary patterns.

4.2.2 Research hypothesis

The statistical null hypothesis will be that, following the intervention, there would be no difference between the control and intervention arms in terms of lipid profile, glucose metabolism, inflammation makers or adiposity. The alternative hypothesis was that there would be differences between the two arms.

5 TRIAL METHODS

5.1 TRIAL DESIGN

The GALIAT study is a community-focused, cluster-randomized clinical trial with two parallel arms designed to examine the effects of a community intervention with a traditional diet on family food behavior. It will be conducted in the primary healthcare setting and led by a health center. The family will form the intervention unit, covering people from childhood to old age. Fieldwork, including the recruitment and follow-up of the participating families, will be performed at the health center.

The main characteristics of this study will be: (1) its focus on the family as the intervention unit; (2) its being based on a diet, the Atlantic diet, congruent with the study area's cultural and gastronomic heritage; (3) the provision of nutritional and gastronomic education (both individual- and community-based); (4) its being conducted in the primary healthcare setting; (5) the participation of six local food companies; and (6) the participation of the City Hall, local media, local companies, a hostelry school, and local restaurants. In summary, the entire community was involved.

Funding was provided by grants from the ERDF-Innterconecta for Galician Program - ITC-20133014 (2013 proposals) and ITC-20151009 (2015 proposals) - managed by the Centre for the Development of Industrial Technology (CDTI), which is part of Spain's Ministry of Economy and Competitiveness. Supplemental foods will be donated, including extra-virgin olive oil (by Aceites Olei), low-fat cheese (by Quescrem), mussels (by Conservas Friscos), vegetables such as turnip greens, cabbage, mushrooms, tomatoes, and mirabelle plums (by Conservas A Rosaleira), red wine (by Bodegas Pazo de Rivas), and white wine (by Bodegas Terras Gauda). None of these companies will participate in the study design, the assessment of results, nor in the writing of the manuscript.

The trial will involve about 250 randomly selected families from a town in Spain's northwest, randomly allocated to either the control or intervention arm of the trial for a period of 6 months. The families in the intervention group will receive educational sessions on food,

diet, and gastronomy and will be provided with written supporting material containing nutritional recommendations and recipes for the preparation of menus. They will also attend cooking classes. Throughout the study period, these families will be provided with a range of foods (free of charge) that form part of the traditional Atlantic diet. The families in the control arm will not take part in any of the above activities, nor will they be provided with any food. Variables will be measured at baseline and six months.

5.2 PROJECT COORDINATION AND LOCATION

The GALIAT study was designed by researchers at the Hospital Clínico Universitario de Santiago de Compostela (The Santiago de Compostela University Hospital) in Spain. Fieldwork, including the recruitment and follow-up of participating families will be performed at the health centre in the rural town of A Estrada. A Estrada has a population of 22,362 and is some 27 km from the aforementioned hospital. This centre was chosen from among several candidates since its personnel have experience of observational studies. Twenty family doctors, three paediatricians and 23 nurses will act as collaborating researchers. In the three weeks before the study began, these persons will be informed of the protocol and work procedures to follow. A further physician, nurse and four to six nutritionists will be employed to lead the field study at the health centre. Their work will involve interviewing the study subjects, the taking of anthropometric data, the measurement of blood pressure, obtaining biological samples, and verifying and transcribing data. The nutritionists will be charged with performing the dietary intervention. All personnel involved in fieldwork will receive theoretical and practical instruction (one month before the study started) on how to normalise work procedures. This will involve trial runs with volunteers subjected to all assessment processes. Daily meetings will focus on the problems detected and their solutions.

Once the trial had begun, daily telephone calls and weekly physical meetings ensured its correct functioning. All fieldwork will be performed in two doctor's offices, plus a room used for the collection of biological samples, provided by the health centre.

5.3 Dietary intervention

The intervention group participants will receive information, motivation, and support to modify their dietary habits according to the Atlantic Diet. To ensure good adherence, a recipe book based on local products has been developed, with detailed recipes created by a chef and evaluated by a team of nutritionists. The nutritionists will provide recommendations to help the intervention subjects adjust their daily and weekly food intakes to meet both the Atlantic Diet and general consensus recommendations. Participants will be encouraged to use the provided recommendations and recipes during the study period, and a day-long cooking course with a chef will also be offered. Additionally, a telephone helpline will be available to address any questions or concerns.

5.4 Randomization

The participating families will be randomly assigned to either the control or intervention arm (1:1) by a person not associated with the study group, using computer-generated random numbers. This was performed after informed consent was signed.

5.5 Sample size and power estimate

The sample size needed to ensure adequate statistical power was determined taking into account the mean cholesterol concentration of the study population (200 mg/dl; inter-person standard deviation 36 mg/dl) and assuming that the dietary intervention would be associated with a reduction of 10 mg/dl. Further assumptions included an intraclass correlation coefficient of 0.25, a family size of 2-4 members, a 90% retention rate, and a 0.05 Type I error rate. Sample size and power estimates were calculated using the Sample Size Shop's GLIMMPSE 2.0 online tool for clustered data.[1] It was determined that a sample size of 250 families would provide a statistical power in excess of 80% for detecting differences between the trial arms.

5.6 Framework

The aim of the GALIAT study is to determine whether the intervention improves clinical outcomes compared to no dietary intervention. The primary and secondary outcomes will be therefore tested for superiority rather than equivalence or inferiority.

5.7 Statistical Interim analyses and trial halting

Since in this study no adverse effects were expected, and given that it lasted 6 months, no guidelines for interruption were planned nor were any interim analyses performed.

5.8 Timing of outcome assessments

The intervention period will last 6 months. The schedule of study procedures is given in Table 1. Table 2 summarizes the information that will be collected, as well as the tests that will be performed, over the study period.

Table 1. Graphical representation of the intervention

Time line	Intervention	Control
Pre- assessment	A B C	A B C
Basal (time 0)	D E F G<br>Randomization<br>H I J<br>K L M	D E F G<br>Randomization<br>H I J
1 week	O	
4 weeks	O P	
7 weeks	O	
10 weeks	O	
3 months	F G L	F G
13 weeks	O	
16 weeks	O	
19 weeks	O	
22 weeks	O	

6 months	F G I J L	F G I J L K 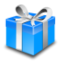
	Measurement of outcomes	

A	Interview between index subject and physician at the health centre. Verbal explanation of the project
B	Provision of written explanation of the project and consent forms (for children/adults/parents & guardians).
C	If participation is accepted, appointment made to see medical team, with family, for baseline assessment, and to provide instructions regarding blood extraction.
D	Check family members meet inclusion criteria but no exclusion criterion
E	Collect consent forms
F	Blood extraction
G	Anthropometric measurements, blood pressure
H	Complete questionnaire on socioeconomic status, check personal and family medical history
I	Complete questionnaire on health-related quality of life, physical activity, sedentarism, diet quality, food consumption patterns, food frequency, alcohol consumption, use of tobacco
J	Hand out of 3-day food record forms
K	Distribute book with educational material and recipes
L	Nutrition education course (30-40 min) provided to individual families by nutritionists; recommendations for adults and children; information on eating five meals per day; preparing menus; explain the Atlantic diet and food pyramid; benefits of physical activity; how to limit

	sedentary activity; how to use the education material provided
M	Diary with food delivery dates (every 3 weeks)
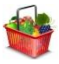	Begin delivery of food packages (adapted for the number of family members)
O	Complete questionnaire on food habits, consumption of delivered foods, physical activity/sedentarism, answering any questions
P	Group session (2 h) by researchers and nutritionists to explain influence of lifestyle on health, changing to a healthier diet, importance of physical activity, characteristics of the traditional Atlantic diet, patterns for designing a healthy diet
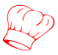	Cooking class led by teaching chefs; recommendations on portion size
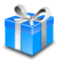	Gift of food basket, educational material and recipe book

Table 2. Outcomes and measurement time points.

	Baseline	3 months	6 months
**Questionnaires & Health Measurements**			
Sociodemographic data	✓		
Health status: personal and family medical history	✓		
Quality of life, (SF-12v2 questionnaire)	✓		✓
Dietary habits, FFQ	✓		✓
3-day food record	✓		✓
Diet quality	✓		✓
Dietary patterns	✓		✓
IPAQ-short	✓		✓
Sedentary behavior	✓		✓
Drinking habits (AUDIT-C questionnaire)	✓		✓
Use of tobacco	✓		✓
**Anthropometry**			
Body weight (kg)	✓	✓	✓
Height (cm)	✓	✓	✓

BMI (kg/m^2)	✓	✓	✓
Waist circumference (cm)	✓		✓
Arm circumference (cm)	✓		✓
Thigh circumference (cm)	✓		✓
Biceps skinfold thickness	✓		✓
Triceps skinfold thickness	✓		✓
Subscapular skinfold thickness	✓		✓
Suprailiac skinfold thickness	✓		✓
Resting pulse and blood pressure	✓		✓
**Biomarkers**			
Total cholesterol	✓	✓	✓
HDL cholesterol	✓	✓	✓
LDL cholesterol	✓	✓	✓
Triglycerides	✓	✓	✓
Albumin	✓	✓	✓
Aspartate aminotransferase	✓	✓	✓
Alanine aminotransferase	✓	✓	✓
Gamma-glutamyl transferase	✓	✓	✓
Creatinine	✓	✓	✓
Urea	✓	✓	✓
Glucose	✓	✓	✓
HbA1c	✓	✓	✓
Insulin	✓		✓
Fructosamine	✓		✓
Leptin	✓		✓
C-reactive protein	✓		✓
Interleukin 6	✓		✓
Tumor necrosis factor α	✓		✓
25-OH cholecalciferol	✓	✓	✓
Thyrotropin	✓		✓
Hemogram	✓	✓	✓

6 STATISTICAL PRINCIPLES

6.1 CONFIDENCE INTERVALS AND *P* VALUES

Significance was set at $P < 0.05$. Results will be expressed with 95% confidence intervals.

6.2 Adherence and Protocol Deviations

Adherence to the protocol will be defined as attendance at scheduled visits. Adherence to changing dietary patterns and lifestyle will be measured through different surveys and questionnaires. The number (and percentage) of patients with protocol deviations will be summarized for each arm. Results will be provided for each arm. Percentage compliance will be provided for each arm.

6.3 Analysis Populations

Both intention-to-treat and per protocol analyses will be performed.

7 Trial Population

7.1 Screening Data

N/A

7.2 Eligibility

The trial inclusion and exclusion criteria are specified in Table 3.

The inclusion criteria to be met by the index subject (male or female) of each family will be: age 18-85 years, and to be part of a family (living together) of at least two members. The other members of the family (either sex) had to be aged 3–85 years.

The following will be deemed reasons for excluding an index subject: alcoholism, undergoing lipid-lowering treatment, pregnancy, cardiovascular disease (ischaemic heart disease, heart failure, peripheral vascular disease, cerebrovascular disease), dementia, or having a predicted survival of less than one year. The exclusion criteria for the family members of the index subject will be the same, except that those following lipid-lowering treatment were included. Finally, for a family to be incorporated into the study, at least two members will meet all inclusion criteria but no exclusion criterion.

Table 3. Inclusion and exclusion criteria.

	Inclusion criteria	Exclusion criteria
**Individual level**		
**Index subject of each family**	Male or female<br>Age 18-85 years<br>To be part of a family (living together) of at least two members	Alcoholism<br>Pregnancy<br>Cardiovascular disease (ischaemic heart disease, heart failure, peripheral vascular disease, cerebrovascular disease)<br>Dementia<br>Predicted survival of less than one year<br>Lipid-lowering treatment
**Other family members**	Male or female<br>Age 3-85 years	Alcoholism<br>Pregnancy<br>Cardiovascular disease (ischaemic heart disease, heart failure, peripheral vascular disease, cerebrovascular disease)<br>Dementia<br>Predicted survival of less than one year
**Cluster (family) level**		
	Two or more members had to meet all inclusion criteria but no exclusion criterion.	

7.3 Recruitment

Families will be chosen via the random selection of index subjects from the National Health System records. At this time, a random selection of 3500 individuals aged 18–85 years was made, stratified by decade. The chosen subjects will be phone in order to confirm their participation in the study. This will provide a list of subjects to whose homes letters explaining the project, and an invitation to take part, will be sent. These subjects will be again called by telephone to reconfirm their participation, to verify that those interested met the inclusion criteria, and to record the size of their families. Those who accepte the invitation to join the study will be given a pre-assessment appointment at which time the project and its aims will be verbally explained, an explanation provided on how subjects were selected, what participation would entail, and the rights of subjects made clear. Documentation providing the details of the project, and consent forms for the whole family, will be also distributed. Those who finally would decide to take part will be invited to return to the health centre, accompanied by their families, for baseline monitoring. The recruitment rate planned will be 10–15 families per week.

To encourage the population to take part, the study will be advertised in the press, on radio and television, and via posters hung in the health center and the A Estrada City Hall. The family doctors involved will help by answering questions about the project.

A CONSORT flow diagram will be prepared, showing the number of people/families screened, those who were eligible, those who take part, who were randomized, who received the allocated treatment, and who withdrew or were lost to follow-up.

7.4 Withdrawal/loss to follow-up

The inclusion of families in the trial naturally ends at 6 months. The directed withdrawal criteria will be the same as the exclusion criteria mentioned above. Not attending programmed appointments and voluntary withdrawal will be also contemplated as withdrawals. Those who will be lost to the study will be not replaced. All reasons for withdrawal/loss will be recorded. This information will be available in the above-mentioned CONSORT diagram.

7.5 Baseline patient characteristics

Baseline characteristics will be described at (1) the family level, i.e., the number of subjects per family, and at (2) the individual level, i.e., age, gender, birthplace, marital status, educational level, employment status, quality of life, physical activity, tobacco and alcohol consumption, personal and family medical history (heart disease, diabetes, high blood pressure, cancer, hypercholesterolemia, age at menarche and menopause) and medication (lipid-lowering and blood pressure-lowering drugs). Health-related quality of life will be assessed using the Spanish v.2.0 of the Short Form 12 Health Survey (SF-12) questionnaire.[2,3] Answers will be interpreted using reference values for Spanish populations.[4]

Adult physical activity will be assessed using the International Physical Activity Questionnaire (short format) [5] from which the metabolic equivalents and hours per week spent in vigorous and moderate activities, and in walking, as described by Craig et al.,[6] will be calculated. This classifies subjects as ‘inactive’, ‘minimally active’, and ‘HEPA active’ (health enhancing physical activity; a high activity category).

For subjects under 18 years of age, the enKid rapid questionnaire (the Krece Plus Short Physical Activity Test) [7] will be used.

8 ANALYSIS

All outcomes will be presented using descriptive statistics - normally distributed data by the mean and standard deviation, and skewed distributions by the median and interquartile range. Binary and categorical variables will presented using numbers and percentages.

The subsections below describe additional analyses.

8.1 OUTCOME DEFINITIONS

8.1.1 Primary outcome

The effects of the intervention on the change in cholesterol levels will be studied via mixed effects models, with 'family' as a random effect, adjusted for age, gender and cholesterol level at baseline. The differences in the change in lipid profile will be also examined.

Results will be expressed as mean differences (95% CI).

8.1.2 Secondary outcomes

Mixed effects models will be also used to assess the effect of the intervention on anthropometric variables, glucose metabolism and inflammatory markers. In these models, different techniques (e.g., logarithmic transformation) will be used when the distribution of the dependent variable was non-Gaussian.

8.1.3 Specific measurement and units

Laboratory results will be reported in conventional units. Anthropometric variables will be recorded in meters and kilograms.

8.1.4 Calculations or transformations used to derive outcomes

The body mass index will be defined as a person's weight in kilograms divided by the square of the person's height in meters (kg/m^2). WHO reference information will be used to classify and interpret data for adults and children.[8]

Percentage body fat will be calculated from the skinfold thickness. There are two equations that allow this transformation, that of Siri [9] (the one chosen) and that of Brozeck.[10] The homeostasis model assessment-estimated insulin resistance (HOMA-IR) index, developed by Matthews et al. [11] will be used to estimate insulin resistance. This is calculated by multiplying fasting plasma insulin in µIU/mL (or mIU/L) by fasting plasma glucose in mg/dL, then dividing by the constant 405.

8.2 DATA VALIDITY AND OUTLIERS

The biomarker results from the laboratory will be automatically downloaded to a database using a computer application. The questionnaires will be filled out by the families in the presence of the nutritionists, who will verify the accuracy of the responses. These data will be independently entered into a database by two operators, and the entries will be compared to detect possible errors. Finally, two researchers will review the database for possible outliers and missing values. In addition, the electronic clinical record of one in every four randomly selected subjects will be reviewed for family history, clinical data, therapeutic prescriptions, and chronic diseases.

8.2.1 Normality

All distributions will be checked for normality. Different treatments will be applied if the distributions were non-Gaussian, e.g., logarithmic transformation, GAMLES models, and others.

8.2.2 Identifying outliers

Box plots, graphs of the residuals against the fitted values, and the Bacon algorithm,[12] will be used to identify outliers.

8.3 MISSING DATA

Missing data will be identified and the reason for their absence investigated. When no reason was determined, these data will be deemed missing at random.

The imputation model will include all the variables, including the dependent variables as well as any other variable that might provide information on the probability of missing data, or about the true values for missing data.

To treat the original data and the multiple imputation data, workflows recommended by StataCorps [13] will be followed.

Multiple imputations will be used to account for subjects with baseline results but missing results at the end of the trial, as well for subjects for whom some data could be not available (biomarkers, anthropometric measurements, surveys, questionnaires, etc.).

Fifty imputations will be performed and the results combined using Rubin's Rules. Imputation will be performed separately for the intervention and control arms.

The distribution of imputed variables will be compared to that of the observed and the complete (i.e., observed and imputed) data.

A sensitivity analysis will be performed to assess the robustness of the results obtained. This involved re-analyzing the primary outcome using techniques not based on multiple imputation (available cases).

8.4 ADVERSE EFFECTS

Although no adverse effects are expected, recording the number (and percentage) of events and their severity will be contemplated for each trial arm.

8.5 STATISTICAL SOFTWARE

Analysis will be performed using R, Stata v.14 and Stata v.16.

9 REFERENCES

1. Kreidler SM, Muller K E, Grunwald G K, et al. GLIMMPSE: Online Power Computation for Linear Models with and without a Baseline Covariate. Journal of Statistical Software. 2013; 54(10).

2. Ware Jr JE, Kosinski M, & Keller SD. A 12-Item Short-Form Health Survey: construction of scales and preliminary tests of reliability and validity. Med Care. 1996; 34 (3): 220-33.
3. Ware JE, Kosinski, M, Turner-Bowker DM, & Gandek B. How to score version 2 of the SF-12® Health Survey (with a supplement documenting Version 1). QualityMetric Incorporated. Lincoln, RI. 2002.
4. Schmidt S, Vilagut G, Garin O, et al. Normas de referencia para el Cuestionario de Salud SF-12 versión 2 basadas en población general de Cataluña. Med Clin. 2012; 139(14): 613-25.
5. The-IPAQ-Group. International Physical Activity Questionnaire (s.f.). (https://sites.google.com/site/theipaq/home.)
6. Craig CL, Marshall AL, Sjöström M, et al. International physical activity questionnaire: 12-country reliability and validity. Med Sci Sports Exerc. 2003; 35(8): 1381-95.
7. Rey-López JP, Vicente-Rodriguez G, Ortega FB, et al. Sedentary patterns and media availability in European adolescents: The HELENA study. Prev. Med. 2010; *51*(1): 50-5.
8. Onis MD, Onyango AW, Borghi E, Siyam A, Nishida C & Siekmann J. Development of a WHO growth reference for school-aged children and adolescents. Bulletin of the World Health Organization. 2007; 85: 660-7.
9. Siri WE. Body composition from fluid spaces and density: analysis of methods. In: Brozeck J, Henschel A, eds. Techniques for measuring body composition. Washington DC: National Academy of Sciences. Natural Resourcer Council. 1961: 223-44.

10. Brožeck J, Grande F, Anderson JT, Keys A. Densitometric analysis of body composition: revision of some quantitative assumptions. Ann NY Acad Sci. 1963; 110 (1): 113-40.
11. Matthews DR, Hosker JP, Rudenski AS, Naylor BA, Treacher DF & Turner RC. Homeostasis model assessment: insulin resistance and β-cell function from fasting plasma glucose and insulin concentrations in man. Diabetologia. 1985; 28(7): 412–9.
12. Weber S. Bacon: An Effective way to Detect Outliers in Multivariate Data Using Stata (and Mata). The Stata Journal. 2010; 10(3): 331–8.
13. Stata multiple-imputation reference manual. Release 16. StataCorps LLC, College Station, Texas.
