# Supplementary material for: Traditional Atlantic Diet and Its Effect on Health and the Environment: A Secondary Analysis of the GALIAT Cluster Randomized Clinical Trial
Source: JAMA Netw Open. 2024 Feb 7;7(2):e2354473. doi: 10.1001/jamanetworkopen.2023.54473 (PMC10851095; doi:10.1001/jamanetworkopen.2023.54473)
Supplement: Supplement 2. — eMethods. eFigure 1. Intervention Components eFigure 2. System Boundaries Considered in the Carbon Footprint Assessment eTable 1. Incidence Rate, Prevalence Rate, and Rate Ratios of Metabolic Syndrome and Its Components by Treatment Group (Per-Protocol Data set) eTable 2. Distribution of MetS Component Score by Treatment Group (Per-Protocol Data set) eFigure 3. Frequency Distribution of Carbon Footprint at Baseline eTable 3. Differences in Dietary Carbon Footprint Between the Intervention and Control Groups (Intention-to-Treat Data set) eTable 4. Differences in Dietary Carbon Footprint Between the Intervention and Control Groups (Per-Protocol Data set) eReferences [file jamanetwopen-e2354473-s002.pdf]

Supplemental Online Content

Cambeses-Franco C, Gude Sampedro F, Benítez-Estévez AJ, et al. Traditional Atlantic diet and its effect on health and the environment: a secondary analysis of the GALIAT cluster randomized clinical trial. *JAMA Netw Open*. 2024;7(2):e2354473. doi:10.1001/jamanetworkopen.2023.54473

eMethods

eFigure 1. Intervention Components

eFigure 2. System Boundaries Considered in the Carbon Footprint Assessment

eTable 1. Incidence Rate, Prevalence Rate, and Rate Ratios of Metabolic Syndrome and Its Components by Treatment Group (Per-Protocol Dataset)

eTable 2. Distribution of MetS Component Score by Treatment Group (Per-Protocol Dataset)

eFigure 3. Frequency Distribution of Carbon Footprint at Baseline

eTable 3. Differences in Dietary Carbon Footprint Between the Intervention and Control Groups (Intention-to-Treat Dataset)

eTable 4. Differences in Dietary Carbon Footprint Between the Intervention and Control Groups (Per-Protocol Dataset)

eReferences

This supplemental material has been provided by the authors to give readers additional information about their work.

© 2024 Cambeses-Franco C et al. *JAMA Network Open.*

eMethods

Procedures

The dietary intervention was based on the Atlantic diet, the traditional dietary pattern in northwestern Spain and Portugal, which is composed of home-cooked local, fresh and minimally processed seasonal products.

The Atlantic diet has several characteristics in common with the Mediterranean diet, such as high consumption of vegetables, fruits, whole grains, beans and olive oil as a key fat source. The Atlantic diet is also characterized by high intake of fish and seafood, starch-based products (mainly potatoes and bread), dried fruits especially chestnuts, milk and cheese, and moderate consumption of meat and wine [1, 2].

The dietary guidance aimed to align food habits with the Atlantic diet's features without a primary focus on energy intake restriction. Dietary advice was tailored to accommodate the individual preferences and nutritional requirements of each participant.

Families in the intervention group participated in three nutrition education sessions (30-40 minutes) at the primary healthcare center at the beginning, and after 3 and 6 months of the study. They also received an educational group session, a cooking class given by a chef in a local restaurant and were provided with written supporting materials, including a recipe book, as well as food baskets (free of charge), delivered every 3 weeks with a variety of local foods characteristic of the traditional Atlantic diet (eFigure 1).

© 2024 Cambeses-Franco C et al. *JAMA Network Open.*

eFigure 1. **Intervention Components**

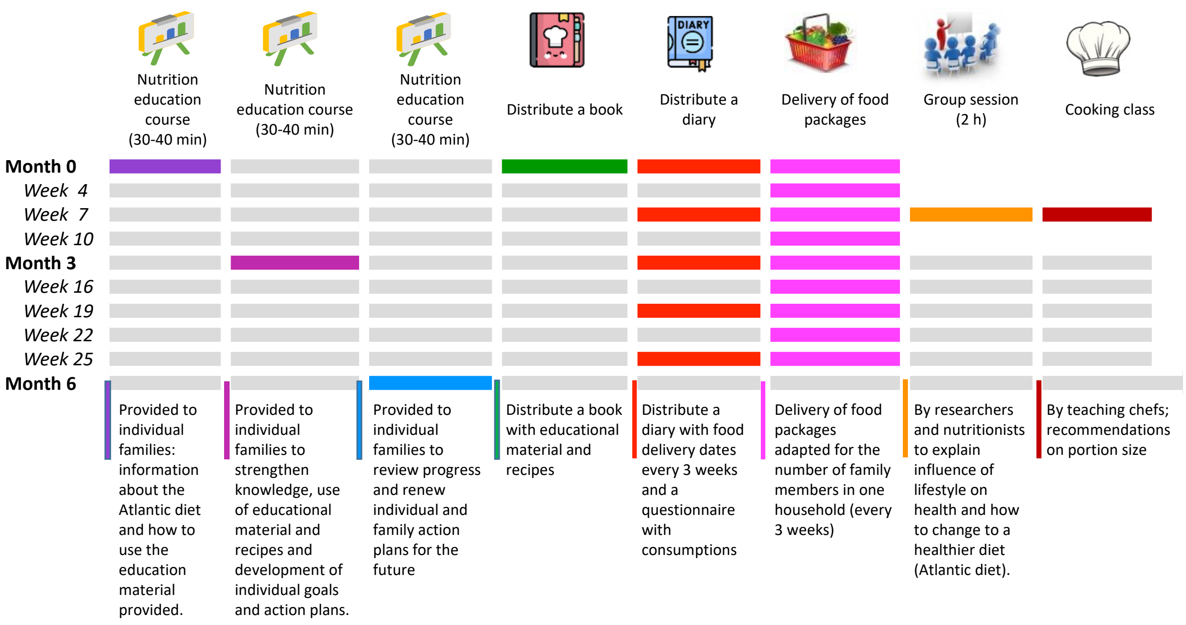

Participants in the control group were advised to continue with their usual lifestyle. Dietary intake and food pattern analysis were assessed in previous articles using a 93-item food frequency questionnaire [3]. Baseline values of dietary patterns were similar between the intervention and control groups, but the intervention group demonstrated significant improvements in healthy patterns [3].

Metabolic variables

Waist circumference was measured at the narrowest point between the bottom of the rib and the top of the iliac crest using a Seca 201 model circumference measuring tape (Hamburg, Germany). Blood pressure was measured using an OMRON M3 automatic

sphygmomanometer after the subjects had been seated for 5 min. Blood was extracted in the morning following a 10–14 h fast. Plasma glucose, high-density lipoprotein (HDL) cholesterol, and triglycerides concentrations were measured using an Advia 2400 Clinical Chemistry System (Siemens Healthcare Diagnostics).

Dietary intake

A 3-day food diary was used to collect dietary intake data. Participants were asked to include information such as foods consumed, brand names, and culinary techniques used. They were advised to provide the weight of all foods consumed, but when that was not feasible, they were to use measurements available at home such as cups and tablespoons. Participants were given a table of equivalencies to assess hand size, household measurement, and weight. Nutritionists verified all records completed by participants in their presence. Dietary intake was analyzed using a professional nutrition analysis software (DIAL V.3.3.5.0, Madrid, Spain) [4].

© 2024 Cambeses-Franco C et al. *JAMA Network Open.*

Carbon footprint methodology

eFigure 2. System Boundaries Considered in the Carbon Footprint Assessment

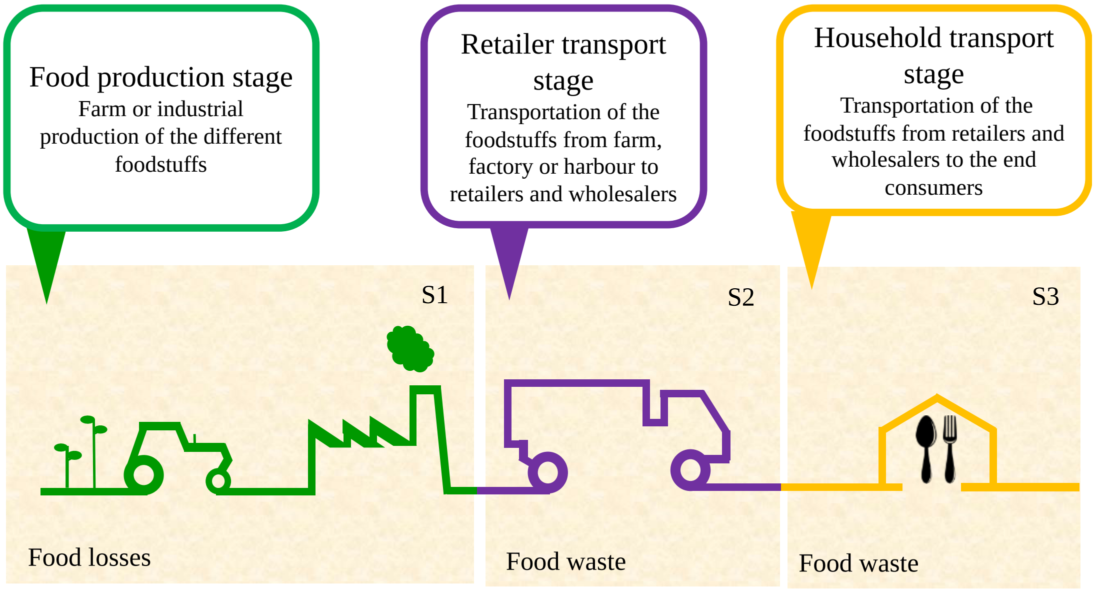

Approach considered for the calculation of the carbon footprint

The food production stage (S1) covers the farm or industrial production of the different food ingredients that constitute the diet of each patient. A systematic literature review was performed to identify GHG emissions associated with the primary production of various food items. We conducted a search on the Scopus and Web of Science databases for studies available in English and published or in press between 2010 and 2023. Additionally, we sought out LCA studies with Spain as the country of reference, utilizing search terms such as 'carbon footprint,' 'Life Cycle,' 'LCA,' and 'LC'. Priority was given to LCA from peer-reviewed journal articles. However, for some specific foodstuffs where information was limited, environmental product declarations and conference papers were also managed. To be consistent with the system boundaries, the LCA studies conformed to the cradle-to-farm gate approach (from raw materials to factory, farm or port), ruling out the carbon footprint contribution of additional stages. A detailed summary of the

© 2024 Cambeses-Franco C et al. *JAMA Network Open.*

sample of 324 food items and their GHG emissions associated were reported in Supplementary Dataset.

The retailer transport stage (S2) includes transportation of the food items from farm, factory or port to retailers and wholesalers. Euro 5 diesel freight lorry (N32 tons) was chosen as transport mode at local and national level. An average truck distance of 60 km was assumed for typical Galician food products [5]. For foodstuffs manufactured in Spain (excluding Galicia), an average distance of 400 km was considered [6]. For international nourishment, sea and land distances between the country of origin and Galician region were calculated. Ecoinvent v3.8. database was used to compute the CO_2 emissions for truck transportation and transoceanic ship [7]. Priority was given to fresh, artisan "100% local" or "zero kilometer" food products. Families bought their groceries in the food market of A Estrada, local supermarkets and village pastry shops. Special attention was paid to the decrease of the amount of food suitable for human consumption along this first stage of the food supply chain (food loss) [8]. Further description of the food losses percentages per food item was reported in the Supplementary Dataset.

The household transport stage (S3) comprises the second distribution channel, from the producer-retailer to the end consumers. Cooking was not included in the system boundaries as in other similar studies available in the literature[7]. Groceries consumed by an individual person in a week (every six days) were acquired and transported from the food store to households over a distance of 3.3 km. A typical diesel passenger vehicle emits about 106 g $CO_2eq \cdot km^{-1}$ [9]. The percentages of food waste, which refer to food thrown away in households, were calculated as detailed in Supplementary Dataset [10].

Carbon footprint calculation: assumptions and limitations

Food production stage (S1):

© 2024 Cambeses-Franco C et al. *JAMA Network Open.*

The great diversity of existing life cycle assessment (LCA) consulted (differenced due to e.g. system boundaries, production method, region specific conditions, agricultural techniques, climate variations…) makes it difficult to estimate the direct carbon footprint scores. Moreover, due to the complexity and wide variety of food products in the managed database, the same carbon footprint was taken for the different varieties of coffee, sausages, spirits, wine, infusions, soft drinks, yoghurt, olives, sugar and breakfast cereals. Differences in greenhouse gas (GHG) emissions between the cuts of beef, chicken and pork were not taking into account either. However, the differentiation between cheeses that were made from different animal species (cow, sheep, goat) was considered. For bread, a distinction was established between white and wholemeal.

Several assumptions were also necessary due to the scarce information for calculating the CF of some particular food items. For them, the GHG emissions were estimated based on data representing a comparable foodstuff. This is the case of saccharin (assimilated to sugar), chard (as spinach), cabbage (as cauliflower and broccoli), kefir (as yoghurt), muesli (as breakfast cereals), custard apple (as avocado), soda and tonic (as soft drink), rice milk (as soy milk), couscous (as wheat seed), loquat (as apricot), dragée (as almond), “callos” Madrid style or other varieties (as beef meat), sausages (as cold meat), chamomile (as tea) and horse meat (as an average value for meat). In all cases, the similarity between the production chains of the foodstuff under study and to which it was assimilate, was evaluated. Moreover, due to the lack of information, the carbon footprint for fruit juice was assimilated to that of its corresponding fruit. For example, apple juice was assimilated to apple. The same consideration was made for strawberry milkshake and cocoa milkshake.

Finally, carbon footprint for hummus, cheesy nachos, kebab, wafer, aioli, and vegetable beef soup was calculated based on its main ingredients according to the Equation 1:

© 2024 Cambeses-Franco C et al. *JAMA Network Open.*

$$CF_{dish} = \sum_{i=1}^{n} CF_i \cdot m_i \qquad (1)$$

Where,

- CF_i is the carbon footprint of each ingredient, in $kgCO_2eq \cdot kg^{-1}$
- m_i is the weight of each ingredient, in kg

Retailer transport stage (S2):

Food waste for high-income and low-income regions was quantified[1]. Due to the absence of specific data for Spain, waste percentages for each food category in the distribution step of the food supply chain for Europe was adopted in this study. Food waste was addressed for the following food categories [8]: cereals; roots & tubers; oilseeds & pulses; fruit & vegetables; meat; fish & seafood and milk & eggs.

Categorization of the large collection of foodstuffs in the database was a complicated task. Some key assumptions were required. Food waste was discarded for soft drinks, liquid beverages (alcoholic and non-alcoholic), liquid chocolate, vinegar, infusions, candy, liquorice, preserved and frozen food. Pastry and bakery food losses were calculated according with the waste percentage of cereal losses. The reason behind this supposition is that flour is the main component of cakes, cookies and pastries. On the other hand, flan is a dairy dessert, and it was included in the milk & eggs category. For fruit juices, jam and strawberry milkshake, the corresponding waste percentage for the fruits and vegetables commodity group was adopted. For coffee, cocoa beans and chocolate powder, their physical characteristics are similar to cereal grains. Consequently, the three were included in the cereals commodity group in order to estimate their weight percentage of food waste at supermarket retail. Curry and soy sauce

© 2024 Cambeses-Franco C et al. *JAMA Network Open.*

were assigned to the fruit & vegetables food group and cheese and béchamel sauce to the milk & eggs food group.

Household transport stage (S3):

Although there may be some economic loss, canned food, candies, liquorice and similar, soft drinks, water, liquid coffee, liquid chocolate, infusions, and frozen food were not considered as quantitative food waste at household level. On the other hand, information about food waste percentages of some food items was scarce. Therefore, some presuppositions were necessary to include all foodstuffs in the database in the food categories established García-Herrero et al. (2018) [10]. This shall apply to alcoholic beverages (included within the group of wine and derivatives); some fruits with unknown waste factor value, as for instance pomegranate or date (considered as "other vegetables" due to the lack of information of food waste percentages for "other fruits"); cocoa (as cereals); bakery products suchlike croissant, cupcake or pastry bun (their food waste factor were comparable to those of the flour due to its primary ingredient is pastry flour); strawberry and cocoa milkshake, cream and chocolate ice-cream (assimilated to its main ingredients, strawberry, cocoa, cream and chocolate, respectively); fruit marmalade (waste at home comparable to its corresponding fruit); cheese sauce, béchamel sauce, soy sauce and curry sauce (as cheese, milk and species, respectively).

© 2024 Cambeses-Franco C et al. *JAMA Network Open.*

Results

Effect of the Galiat intervention on MetS and its components

eTable 1. Incidence Rate, Prevalence Rate, and Rate Ratios of Metabolic Syndrome and Its Components by Treatment Group (Per-Protocol Dataset)

Criteria	Control group		Intervention group		RRs (95% CI)	P-value
	Baseline (%, 95% CI)	6 months (%, 95% CI)	Baseline (%, 95% CI)	6 months (%, 95% CI)		
Abdominal obesity	40.3 (34.4-46.6)	42.3 (36.3-48.6)	52.6 (46.6-58.5)	47.8 (41.9-53.8)	0.90 (0.81-0.99)	*.04*
Hypertriglyceridaemia	20.2 (15.6-25.6)	18.5 (14.2-23.9)	14.1 (10.4-18.8)	13.3 (9.8-17.9)	0.89 (0.64-1.24)	*.49*
Low HDL-cholesterol	17.7 (13.5-23.0)	26.2 (21.1-32.1)	18.5 (14.3-23.6)	21.1 (16.6-26.4)	0.79 (0.63-0.99)	*.04*
High blood pressure	41.9 (35.9-48.2)	43.1 (37.1-49.3)	50.0 (44.1-55.9)	43.7 (37.9-49.7)	0.86 (0.74-0.99)	*.04*
Hyperglycaemia	17.7 (13.5-23.0)	14.9 (11.0-19.9)	17.8 (13.7-22.8)	14.4 (10.7-19.2)	0.97 (0.72-1.31)	*.85*
MetS	19.8 (15.3-25.2)	19.0 (14.5-24.3)	21.9 (17.3-27.2)	18.1 (14.0-23.2)	0.89 (0.68-1.15)	*.35*
MetS incidence rate		6.0 (3.5-10.3)		2.8 (1.3-6.2)	0.40 (0.15-1.06)	*.07*

Values used from the intention-to-treat data set. Figures are prevalences and incidence in percentages with 95% confidence intervals (95%CI). RRs are rate ratios adjusted by sex and age, and were estimated using Poisson regression models for incident cases of MetS per 6 months.
Criteria from the Adult Treatment Panel III for the definition of metabolic syndrome: abdominal obesity, defined by waist circumference > 102 cm in males or > 88 cm in females; hypertriglyceridaemia, defined by fasting serum triglycerides ≥ 150 mg/dL; low high-density lipoprotein (HDL)-cholesterol levels, defined by fasting HDL-cholesterol < 40 mg/dL in males or < 50 mg/dL in females; high blood pressure, defined by blood pressure ≥ 130/85 mmHg or current anti-hypertensive medication use; and hyperglycaemia, defined by fasting blood glucose ≥ 110 mg/dL or current anti-diabetic therapy. Individuals meeting at least three of these criteria were considered to have metabolic syndrome.

© 2024 Cambeses-Franco C et al. *JAMA Network Open.*

eTable 2. Distribution of MetS Component Score by Treatment Group (Per-Protocol Dataset)

Number of fulfilled components of MetS	Control group		Intervention group		RR (95% CI)	P-value
	Baseline (%, 95% CI)	6 months (%, 95% CI)	Baseline (%, 95% CI)	6 months (%, 95% CI)		
0	0.33 (0.28-0.39)	0.31 (0.25-0.37)	0.25 (0.20-0.31)	0.32 (0.27-0.38)	0.57 (0.40-0.82)	.002
1	0.24 (0.19-0.30)	0.21 (0.17-0.27)	0.30 (0.25-0.36)	0.25 (0.20-0.31)		
2	0.23 (0.19-0.29)	0.29 (0.24-0.35)	0.23 (0.18-0.28)	0.25 (0.20-0.30)		
3	0.13 (0.10-0.18)	0.11 (0.08-0.15)	0.12 (0.09-0.16)	0.10 (0.07-0.14)		
4	0.05 (0.03-0.08)	0.07 (0.05-0.11)	0.09 (0.06-0.13)	0.06 (0.04-0.10)		
5	0.02 (0.01-0.04)	0.01 (0.00-0.03)	0.01 (0.01-0.04)	0.03 (0.01-0.05)		

Figures are proportional prevalences and 95% confidence intervals (95%CI). POR is proportional Odds Ratio with 95%CI. Ordered logistic regression models was performed to estimate POR and 95%CI, adjusted by age and sex.

Effect of the Galiat intervention on carbon footprint

The carbon footprint data exhibited a normal distribution (eFigure 3).

eFigure 3. Frequency Distribution of Carbon Footprint at Baseline

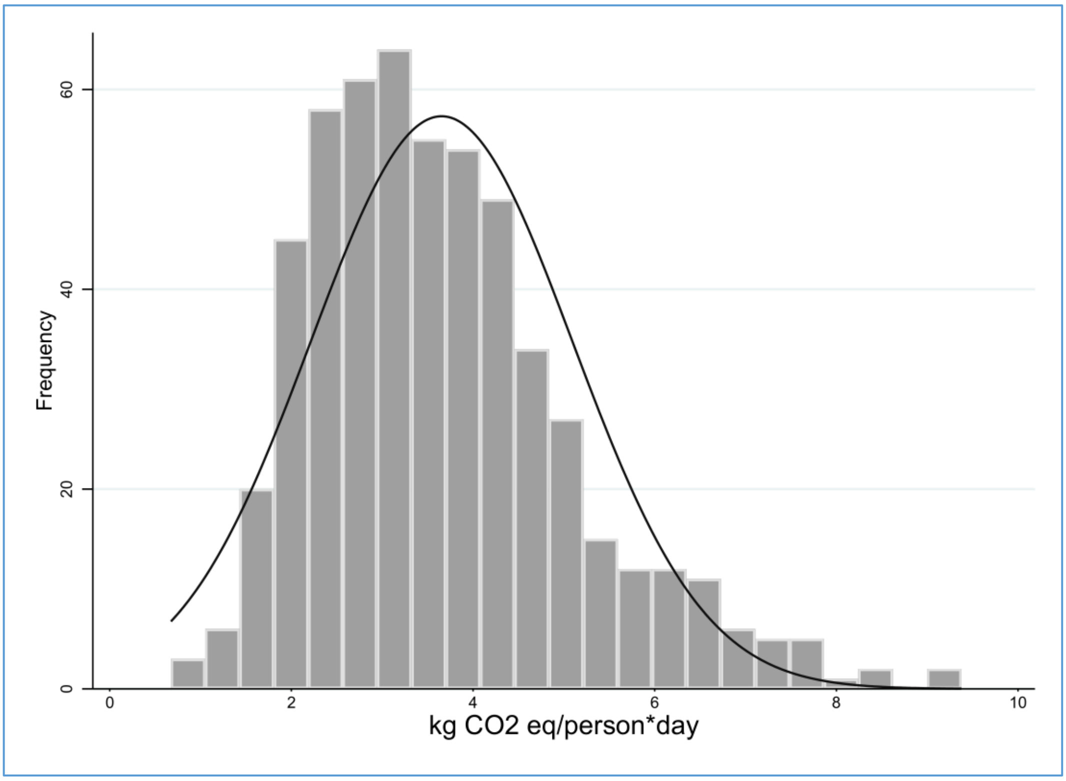

© 2024 Cambeses-Franco C et al. *JAMA Network Open.*

eTable 3. Differences in Dietary Carbon Footprint Between the Intervention and Control Groups (Intention-to-Treat Dataset)

	Control (n=287)		Intervention (n=287)		Multilevel mixed-effects linear regression (n=574)		
	**Baseline**	**6 months**	**Baseline**	**6 months**	**Coefficient intervention group**	**p-value**	**ICC**
**Carbon footprint**	3.71 ± 1.55	3.56 ± 1.50	3.60 ± 1.44	3.38 ± 1.39	-0.17 (-0.45-0.10)	*.22*	0.45

Carbon footprint expressed in kg CO_2 eq·person^{-1}·day^{-1}. Baseline and 6-month data are presented as mean and standard deviation. ICC intraclass coefficient.

eTable 4. Differences in Dietary Carbon Footprint Between the Intervention and Control Groups (Per-Protocol Dataset)

	Control (n=238)		Intervention (n=241)		Multilevel mixed-effects linear regression (n=479)		
	**Baseline**	**6 months**	**Baseline**	**6 months**	**Coefficient intervention group**	**p-value**	**ICC**
**Carbon footprint**	3.70 ± 1.52	3.54 ± 1.37	3.64 ± 1.43	3.37 ± 1.23	-0.17 (-0.46-0.12)	*.24*	0.51

Carbon footprint expressed in kg CO_2 eq·person^{-1}·day^{-1}. Baseline and 6-month data are presented as mean and standard deviation. ICC intraclass coefficient.

© 2024 Cambeses-Franco C et al. *JAMA Network Open.*

Statistical Models and Analysis Details (Stata Outputs)

Rate ratio for new MetS in the intervention group vs. controls adjusted for age and gender (Intention-to-treat data set)

```
. mi estimate: glm sm_diag3 sexo edad grupo if sm_diag1==0, fam(poisson) link(log) nolog vce(robus) eform

Multiple-imputation estimates                   Imputations       =         30
Generalized linear models                       Number of obs     =        457
                                                Average RVI       =     0.0000
                                                Largest FMI       =     0.0000
DF adjustment:   Large sample                   DF:     min       =   4.80e+62
                                                        avg       =   4.80e+62
                                                        max       =          .
Model F test:       Equal FMI                   F(   3, 1.1e+64)  =       4.98
Within VCE type:       Robust                   Prob > F          =     0.0019

------------------------------------------------------------------------------
    sm_diag3 |      Coef.   Std. Err.      t    P>|t|     [95% Conf. Interval]
-------------+----------------------------------------------------------------
        sexo |   .6049632   .4016536     1.51   0.132    -.1822634     1.39219
        edad |   .0340553   .0138889     2.45   0.014     .0068336     .061277
       grupo |  -1.137757   .4630406    -2.46   0.014      -2.0453   -.2302144
       _cons |  -4.478517   .7389667    -6.06   0.000    -5.926865   -3.030169
------------------------------------------------------------------------------

.
. display =exp(-1.137757)
.32053718

. display =exp(-2.0453)
.12934138

. display =exp(-.2302144)
.79436327
```

Rate ratio for new MetS in the intervention group vs. controls adjusted for age and gender (Per protocol data set)

```
. glm sm_diag3 ib0.sexo edad ib0.grupo if sm1!=. & sm3!=. & sm_diag1==0, fam(poisson) link(log) nolog vce(robus) eform

Generalized linear models                         Number of obs   =        410
Optimization     : ML                             Residual df     =        406
                                                  Scale parameter =          1
Deviance         =  101.4396413                   (1/df) Deviance =   .2498513
Pearson          =  383.2985525                   (1/df) Pearson  =   .9440851

Variance function: V(u) = u                       [Poisson]
Link function    : g(u) = ln(u)                   [Log]

                                                  AIC             =   .3547308
Log pseudolikelihood = -68.71982067               BIC             =   -2341.12

------------------------------------------------------------------------------
             |               Robust
    sm_diag3 |        IRR   Std. Err.      z    P>|z|     [95% Conf. Interval]
-------------+----------------------------------------------------------------
        sexo |
         man |   2.169596   .9896968     1.70   0.090     .8873384    5.304793
        edad |    1.03708   .0162127     2.33   0.020     1.005785    1.069348
             |
       grupo |
Intervention |   .4029452     .19868    -1.84   0.065     .1533009    1.059126
       _cons |   .0075969   .0060869    -6.09   0.000     .0015799    .0365297
------------------------------------------------------------------------------
Note: _cons estimates baseline incidence rate.
```

© 2024 Cambeses-Franco C et al. *JAMA Network Open.*

Rate ratio for prevalence of MetS in the intervention group vs. controls adjusted for age and gender (Intention-to-treat data set)

```
. mi estimate: glm sm_diag3 ib1.sm_diag1 ib0.sexo edad ib0.grupo, fam(poisson) link(log) nolog vce(robus) eform

Multiple-imputation estimates                   Imputations       =         30
Generalized linear models                       Number of obs     =        574
                                                Average RVI       =     0.0000
                                                Largest FMI       =     0.0000
DF adjustment:   Large sample                   DF:     min       =          .
                                                        avg       =          .
                                                        max       =          .
Model F test:       Equal FMI                   F(   4,      .)   =      51.83
Within VCE type:       Robust                   Prob > F          =     0.0000

------------------------------------------------------------------------------
    sm_diag3 |      Coef.   Std. Err.      t    P>|t|     [95% Conf. Interval]
-------------+----------------------------------------------------------------
  0.sm_diag1 |  -2.498178    .234497   -10.65   0.000    -2.957784   -2.038572
             |
        sexo |
         man |   .1179297   .1331356     0.89   0.376    -.1430113    .3788708
        edad |   .0121802    .005481     2.22   0.026     .0014378    .0229227
             |
       grupo |
Intervention |  -.1971967   .1285203    -1.53   0.125    -.4490918    .0546985
       _cons |  -1.001263    .335664    -2.98   0.003    -1.659153   -.3433739
------------------------------------------------------------------------------

.
. display =exp(-.1971967)
.82102912

. display =exp(-.4490918)
.63820751

. display =exp(.0546985)
1.0562221
```

Rate ratio for prevalence of MetS in the intervention group vs. controls adjusted for age and gender (Per protocol data set)

```
. glm sm_diag3 ib0.sexo edad ib0.grupo if sm1!=. & sm3!=. & sm_diag1==0, fam(poisson) link(log) nolog vce(robus) eform

Generalized linear models                         Number of obs   =        410
Optimization     : ML                             Residual df     =        406
                                                  Scale parameter =          1
Deviance         =  101.4396413                   (1/df) Deviance =   .2498513
Pearson          =  383.2985525                   (1/df) Pearson  =   .9440851

Variance function: V(u) = u                       [Poisson]
Link function    : g(u) = ln(u)                   [Log]

                                                  AIC             =   .3547308
Log pseudolikelihood = -68.71982067               BIC             =   -2341.12

------------------------------------------------------------------------------
             |               Robust
    sm_diag3 |        IRR   Std. Err.      z    P>|z|     [95% Conf. Interval]
-------------+----------------------------------------------------------------
        sexo |
         man |   2.169596   .9896968     1.70   0.090     .8873384    5.304793
        edad |    1.03708   .0162127     2.33   0.020     1.005785    1.069348
             |
       grupo |
Intervention |   .4029452     .19868    -1.84   0.065     .1533009    1.059126
       _cons |   .0075969   .0060869    -6.09   0.000     .0015799    .0365297
------------------------------------------------------------------------------
Note: _cons estimates baseline incidence rate.
```

© 2024 Cambeses-Franco C et al. *JAMA Network Open.*

Effect of intervention on the number of MetS risk factors (Intention-to-treat data set)

```
. mi estimate: ologit sm3 sm1 ib0.sexo edad ib0.grupo

Multiple-imputation estimates                 Imputations       =         30
Ordered logistic regression                   Number of obs     =        574
                                              Average RVI       =     0.0000
                                              Largest FMI       =     0.0000
DF adjustment:   Large sample                 DF:     min       =   1.48e+62
                                                      avg       =   1.48e+62
                                                      max       =          .
Model F test:       Equal FMI                 F(   4, 8.1e+63)  =      96.67
Within VCE type:          OIM                 Prob > F          =     0.0000
```

sm3	Coef.	Std. Err.	t	P>|t|	[95% Conf.	Interval]
sm1	2.175924	.1165821	18.66	0.000	1.947427	2.404421
sexo						
man	.2708142	.172639	1.57	0.117	-.067552	.6091804
edad	.0177044	.0060128	2.94	0.003	.0059196	.0294892
grupo						
Intervention	-.537541	.1723919	-3.12	0.002	-.8754228	-.1996591
/cut1	1.743191	.2950963			1.164812	2.321569
/cut2	3.964724	.3335458			3.310986	4.618461
/cut3	6.995174	.430026			6.152339	7.83801
/cut4	8.89502	.4952299			7.924387	9.865652
/cut5	11.67362	.6518764			10.39597	12.95127

```
. display ==exp(-.537541)
.58418299

. display ==exp(-.8754228)
.41668581

. display ==exp(-.1996591)
.81900991
```

© 2024 Cambeses-Franco C et al. *JAMA Network Open.*

Effect of intervention on the number of MetS risk factors (Per protocol data set)

```
. ologit sm3 sm1 ib0.sexo edad ib0.grupo if sm_diag1!=. & sm_diag3!=.

Iteration 0:   log likelihood = -797.01729
Iteration 1:   log likelihood = -542.47886
Iteration 2:   log likelihood =    -496.21
Iteration 3:   log likelihood = -494.55525
Iteration 4:   log likelihood = -494.54868
Iteration 5:   log likelihood = -494.54868

Ordered logistic regression                     Number of obs     =        518
                                                LR chi2(4)        =     604.94
                                                Prob > chi2       =     0.0000
Log likelihood = -494.54868                     Pseudo R2         =     0.3795
```

sm3	Coef.	Std. Err.	z	P>|z|	[95% Conf.	Interval]
sm1	2.211633	.1241774	17.81	0.000	1.96825	2.455017
sexo						
man	.1680473	.1835459	0.92	0.360	-.1916961	.5277907
edad	.0198391	.0063702	3.11	0.002	.0073539	.0323244
grupo						
Intervention	-.5569498	.1831726	-3.04	0.002	-.9159615	-.1979381
/cut1	1.892966	.3184375			1.26884	2.517092
/cut2	4.039085	.3581114			3.3372	4.740971
/cut3	7.206805	.467025			6.291453	8.122157
/cut4	9.133485	.5355007			8.083923	10.18305
/cut5	11.81307	.6865964			10.46736	13.15877

```
. display ==exp(-.5569498)
.57295403

. display ==exp(-.9159615)
.40013171

. display ==exp(-.1979381)
.82042064
```

© 2024 Cambeses-Franco C et al. *JAMA Network Open.*

eReferences

1. Calvo-Malvar M, Leis R, Benítez-Estévez AJ, Sánchez-Castro J, Gude F. A randomised, family-focused dietary intervention to evaluate the Atlantic diet: the Galiat study protocol. *BMC Public Health.* 2016;16:820.

2. Vaz Velho M, Pinheiro R, Rodrigues AS. The Atlantic diet—origin and features. *Int J Food Stud.* 2016;5:106-19.

3. Calvo-Malvar M, Benítez-Estévez AJ, Leis R, Sánchez-Castro J, Gude F. Changes in Dietary Patterns through a Nutritional Intervention with a Traditional Atlantic Diet: The Galiat Randomized Controlled Trial. *Nutrients*. 2021; 13(12):4233.

4. Ortega RM, López AM, Carvajales PA, Requejo AM, Aparicio A, Molinero LM. Programa Dial v. 3.3.5.0. 2016.

5. Esteve-Llorens X, Darriba C, Moreira MT, Feijoo G, González-García S. Towards an environmentally sustainable and healthy Atlantic dietary pattern: Life cycle carbon footprint and nutritional quality. *Sci Total Environ*. 2019; 646: 704–15.

6. Castañé S, Antón A. Assessment of the nutritional quality and environmental impact of two food diets: A Mediterranean and a vegan diet. *J Clean Prod.* 2017; 167: 929–37.

7. Werner, G., Bauer, C., Steubing, B., Reinhard, J., Moreno-Ruiz, E., Weidema, B.. The ecoinvent database version 3 (part I): overview and methodology. *Int. J. Life Cycle Assess.* 2016, 21, 1218-1230.

8. Gustavsson J, Cederberg C, Sonesson U, Emanuelsson A. The methodology of the FAO study : "Global Food Losses and Food Waste - extent, causes and prevention"-FAO, 2011. 2013 https://www.diva-portal.org/smash/get/diva2:944159/FULLTEXT01.pdf.

9. Batlle-Bayer L, Bala A, García-Herrero I, et al. The Spanish Dietary Guidelines: A potential tool to reduce greenhouse gas emissions of current dietary patterns. *J Clean Prod.* 2019; 213: 588–98.

10. Garcia-Herrero I, Hoehn D, Margallo M, et al. On the estimation of potential food waste reduction to support sustainable production and consumption policies. *Food Policy*. 2018; 80: 24–38.

© 2024 Cambeses-Franco C et al. *JAMA Network Open.*
