# Supplementary material for: Traditional Atlantic Diet and Its Effect on Health and the Environment: A Secondary Analysis of the GALIAT Cluster Randomized Clinical Trial
Source: JAMA Netw Open. 2024 Feb 7;7(2):e2354473. doi: 10.1001/jamanetworkopen.2023.54473 (PMC10851095; doi:10.1001/jamanetworkopen.2023.54473)
Supplement: Supplement 4. — Data Sharing Statement [file jamanetwopen-e2354473-s004.pdf]

Data Sharing Statement

Cambeses-Franco. Traditional Atlantic Diet and Its Effect on Health and the Environment. *JAMA Netw Open*. Published February 07, 2024. doi:10.1001/jamanetworkopen.2023.54473

Data
Data available: Yes
Data types: Deidentified participant data
How to access data: The datasets generated and analyzed during the current study will be made available through a publicly accessible repository on publication at the Runa Digital Repository (runa.sergas.gal). To gain access, data requestors will need to sign a data access agreement. Proposals should be directed to alfonsojavier.benitez.estevez@sergas.es and/or francisco.gude.sampedro@sergas.es.
When available: With publication

Supporting Documents
Document types: None

Additional Information
Who can access the data: Researchers whose proposed use of the data has been approved
Types of analyses: Meta-analysis, systematic reviews, reviews.
Mechanisms of data availability: After approval of a proposal
